# Effect-enhancing and toxicity-reducing effects of Chaihu Jia Longgu Muli decoction in the treatment of multimorbidity with depression: a systematic review and meta-analysis

**DOI:** 10.1080/13880209.2023.2228356

**Published:** 2023-07-13

**Authors:** Xurui Jia, Jie Chen, Ruiou Huang, Dawei Wang, Xing Wang

**Affiliations:** a Affiliated Hospital of Integrated Traditional Chinese and Western Medicine, Nanjing University of Chinese Medicine, Nanjing, China; b School of Basic Medicine and Clinical Pharmacy, China Pharmaceutical University, Nanjing, China; c Brain Hospital Affiliated to Nanjing Medical University, Nanjing, China; d Children’s Hospital of Nanjing Medical University, Nanjing, China; e Jiangsu Health Vocational College, Nanjing, Jiangsu Province China

**Keywords:** Traditional Chinese medicine, antidepressant, combination therapy

## Abstract

**Context:**

Chaihu Jia Longgu Muli Decoction (CLMD) is a traditional Chinese medicine for treating depression.

**Objective:**

This study investigated the effect of CLMD combined with antidepressants on multimorbidity with depression (MMD).

**Method:**

Published randomized controlled trials were collected from PubMed, Web of Science, Chinese National Knowledge Infrastructure, China Science and Technology Journal, Wanfang and China Biomedical Literature Service System Databases. Participants were divided into study groups (CLMD combined with antidepressants) and control groups (antidepressants). RevMan5.4 software was used for data analysis.

**Results:**

Hamilton’s Depression Scale score was significantly lower (MD = −5.62, 95%CI [−5.86, −5.37], *p* < 0.00001), and the effective rate was significantly higher (RR = 1.23, 95%CI [1.17, 1.29], *p* < 0.00001) in study groups. National Institute of Health Stroke Scale score and Pittsburgh Sleep Quality Index score of study groups were significantly lower (MD = −2.82, 95%CI [−3.84, −1.81], *p* < 0.00001; MD = −2.26, 95%CI [−3.19, −1.34], *p* < 0.00001). 5-HT, DA, NE and IL-1β level were significantly lower (SMD = 1.99, *p* = 0.003; SMD = 1.99, *p* < 0.00001; SMD = 0.86, *p* < 0.00001; MD = −14.64, *p* = 0.003) in study groups. Adverse reaction rate in study groups was significantly lower (RR = 0.47, 95%CI [0.24, 0.91], *p* = 0.03). The gastrointestinal tract and autonomic nervous system disorders were reduced in study groups.

**Discussion and conclusions:**

CLMD combined with antidepressants enhances the effect of antidepressants and reduces their adverse reactions, performing a synergistic effect; it may be considered as an effective option in the treatment of MMD.

## Introduction

Multimorbidity (Pietzner et al. 2021) describes a disease state in which multiple chronic diseases coexist. According to World Health Organization (WHO) (Moussavi et al. 2007), the incidence of depression in people with at least one chronic disease is about three times higher than that in the normal population. Depression has a higher incidence in patients with chronic diseases: cardiovascular disease (17–27%), diabetes (11–31%), arthritis (10–24%). It was suggested that the probability of depression increases by 45% for each additional chronic complication (Read et al. 2017). The study of Pietzner et al. (2021) revealed the characteristics of a high correlation among non-communicable diseases (NCDS), and the same metabolites may also be shared by different NCDS. Depression and comorbidities may have the same mechanism (Pietzner et al. 2021), which indicated the significance of multimorbidity with depression (MMD) research.

MMD is an important feature of chronic disease, representing bio-psycho-social medical model. Depression coexists with many chronic diseases, for instance, cancer, stroke, coronary heart disease, diabetes, vertigo disease, anxiety, insomnia, and immune rheumatism. We should attach importance to the concept of holism and change the disease-cognizing concept from disease-centered to patient-centered, which is consistent with the concept of holism in traditional Chinese medicine (TCM), underlining the correspondence between human body and natural environment and psychosomatic oneness.

The second-generation antidepressants including selective serotonin reuptake inhibitors (SSRIs), serotonin norepinephrine reuptake inhibitors (SNRIs), noradrenergic and specific serotonergic antidepressants (NaSSA), are not only recommended for depressive orders treatment (Guo et al. 2020), but also applied for MMD in clinical practice considering the coexisting diseases. The pathogenesis of depression is complex, while the existing western antidepressants have a single therapeutic mechanism. Therefore, starting from multiple targets, the western medicine-western medicine, western medicine-TCM, TCM-TCM combined treatment is becoming a new method in clinics (Liu et al. 2020).

Current studies are mostly based on one certain disease, while multiple chronic diseases are more common in patients. There are different degrees of correlation between various diseases, and one certain disease can be the dominant disease at one certain stage, which brings uncertainty about clinical condition to patients with multimorbidity. The coexistence of multiple diseases with depression is a common problem, for which clinical research targeting at various MMD has great significance in chronic disease management. So, it’s urgent to explore the treatment options of MMD.

Currently, the preferred treatment for MMD is antidepressants, which are deficient in specificity for the coexisting disease. In addition, the limitations like delayed onset of efficacy, drug tolerance and resistance, drug interactions and side effects are common in antidepressants. Traditional Chinese medicine has unique advantages in psychosomatic diseases such as depression. Chaihu Jia Longgu Muli Decoction (CLMD) is a classic compound prescription for depression, known as ‘special formula for antidepressant’, which is widely used in emotional diseases, including depression, insomnia, anxiety, menopausal syndrome. Preliminary clinical and basic studies (Wang, Zou, et al. 2019; Wang, Chen, et al. 2019) have confirmed that CLMD has clear antidepressant effects and rapid antidepressant onset in depression and MMD. Setting MMD as a breakthrough point, this study aims to discuss the application of CLMD in clinical practice by systematic review and meta-analysis to support the clinical value of compound TCM in MMD and to provide research ideas for future studies.

## Methods

This systematic review and meta-analysis is reported according to the Preferred Reporting Items for Systematic Reviews and Meta-Analyses (PRISMA) Statement (Page et al. 2021) and is registered in PROSPERO. The protocol and registration information are available at PROSPERO (https://www.crd.york.ac.uk/prospero/display_record.php?ID=CRD42022341499).

### Search strategy

Chinese National Knowledge Infrastructure (CNKI), VIP Database, Wanfang Database, China Biomedical Literature Service System (CBM), PubMed, Web of Science were searched by the following strategy: ‘Chaihu Jia Longgu Muli Decoction’ plus ‘Depression’ plus ‘Multimorbidity’ in both Chinese and English term [title/theme]. The publication time was from the setup of database to June 26th, 2022.

### Inclusion and exclusion criteria

#### Inclusion criteria

Included literature satisfied the following criteria:

1. Research type: Randomized controlled clinical studies (RCTs) of CLMD combined with antidepressants.
2. Disease type: Subjects from literature should meet the diagnostic criteria for depression (Chinese Society of Psychiatry 2001) and were complicated with one or more major physical disease.
3. Interventions: Control group (antidepressants alone) vs. Study group (antidepressants combined with oral CLMD). Antidepressants included selective serotonin reuptake inhibitors (SSRIs), serotonin norepinephrine reuptake inhibitors (SNRIs), monoamine oxidase inhibitors (MAOIs), tricyclics (TCAs), norepinephrine dopamine reuptake inhibitors (NDRIs). The medicinal plants used to prepare CLMD were Bupleuri Radix [*Bupleurum chinense* DC. or *Bupleurum scorzonerifolium* Willd. (Umbelliferae) radix; Chaihu in Chinese], Os Draconis [*Fossilia Ossis* Mastodi; Longgu in Chinese], Scutellariae Radix [*Scutellaria baicalensis* Georgi (Labiatae) radix; Huangqin in Chinese], Rhizoma Zingiberis Recens [*Zingiber officinale* Rosc. (Zingiberaceae) fresh Rhizoma; Shengjiang in Chinese], Ginseng Radix et Rhizoma [*Panax ginseng* C. A. Mey. (Araliaceae) radix and rhizoma; Renshen in Chinese], Cinnamomi Ramulus [*Cinnamomum cassia* Presl (Subfam. Lauroideae) ramulus; Guizhi in Chinese], Poria [*Poria cocos* (Schw.) Wolf (Polyporaceae) dry sclerotia; Fuling in Chinese], Pinelliae Rhizoma [*Pinellia ternate* (Thunb.) Breit. (Araceae) rhizoma; Banxia in Chinese], Rhei Radix et Rhizoma [*Rheum palmatum* L., *Rheum tanguticum* Maxim. ex Balf. or *Rheum officinale* Baill. (Polygonaceae) radix and rhizoma; Dahuang in Chinese], Ostreae Concha [*Ostrea gigas* Thunberg, *Ostrea talienwhanensis* Crosse or *Ostrea rivularis* Gould (Ostreidae) concha; Muli in Chinese], and Fructus Jujubae [*Ziziphus jujuba* Mill. (Rhamnaceae) fructus; Dazao in Chinese].

#### Exclusion criteria

The following literature were excluded:

1. Studies which were not RCTs.
2. Animal trials.
3. Expert experience or reviews.
4. Studies with incomplete data, or outcome indicators of which could hardly be extracted.
5. Studies rated as low quality after reading.
6. Studies with high-risk protocol.

### Outcome indicators

#### Primary indicators

- Total effective rate: The effective rate was judged by the reduction rate of Hamilton’s Depression Scale score before and after the treatment. According to reduction rate, the outcome was divided into following types: clinical cure (reduction rate >75%), markedly effective (reduction rate >50%), effective (reduction rate ≥25%), ineffective (reduction rate <25%). Total effective rate = (clinical cure number + markedly effective number + effective number)/total number × 100%.
- Hamilton’s Depression Scale (HAMD) score.

#### Secondary indicators

- National Institutes of Health stroke scale (NIHSS) score.
- Pittsburgh sleep quality index (PSQI) score.
- Monoamine transmitter level (5-HT, DA and NE), cytokine level (TNF-α, IL-1β).
- Incidence of adverse reaction.

HAMD score can not only reflect the disease status of depressed patients, but also reflect the change of treatment effect in time dimension. Judged by the reduction of HAMD score, total effective rate can reflect the overall difference in treatment effect between the study group and the control group, which was widely used in published articles. Monoamine transmitter and cytokine level was proved to be highly correlated with patients’ depressive states (Dahl et al. 2014; Dean and Keshavan 2017). In terms of coexisting disease treatment, PSQI and NIHSS score can respectively assess the sleeping quality and neurological function of patients. The incidence of adverse reaction was record in some publications, which can be used to assess the toxicity of combination treatment.

### Data extraction

Two researchers independently screened literature according to inclusion and exclusion criteria independently. Literature information (titles, author name and publication time), research information (subject multimorbidity types, interventions, subject cases, outcome indicators) and crucial data were extracted from literature. The third researcher would get involved to evaluate the controversial data and content.

### Quality assessment

Risk of bias assessment tool recommended by Cochrane manual (Higgins et al. 2022) was used to evaluate the bias risk in following aspects: random sequence generation, allocation concealment, blinding of participants and personnel, blinding of outcome data, incomplete data, selective reporting, and other bias. Each study was supposed to be rated as ‘high risk of bias’, ‘unclear of bias’ or ‘low risk of bias’.

### Statistical analysis

For discontinuous variables such as total effective rate, relative risk (RR) was used for analysis. For continuous variables with same measurement units, the weighted mean difference (MD) was used for analysis. While for continuous variables with different measurement units, the standard mean difference (SMD) was used for analysis. Effect estimate was presented with 95% confidence interval (95% CI). Statistical heterogeneity among was evaluated using chi-square test and was quantified by *I*^2^. For *I*^2^ values great than 50% regarded as being indicative of significant heterogeneity, a random-effect model was used for analysis. For results with no significant heterogeneity, a fixed-effect model was used to estimate the effect. We used RevMan5.4 for data integration and statistical analysis. We conducted sensitivity analysis to explore the effect of single study on overall effect. And subgroup analysis would be performed to explore the heterogeneity between studies and the influence of different intervention factors on the results.

## Results

### Characteristics of studies included

**Figure 1.**
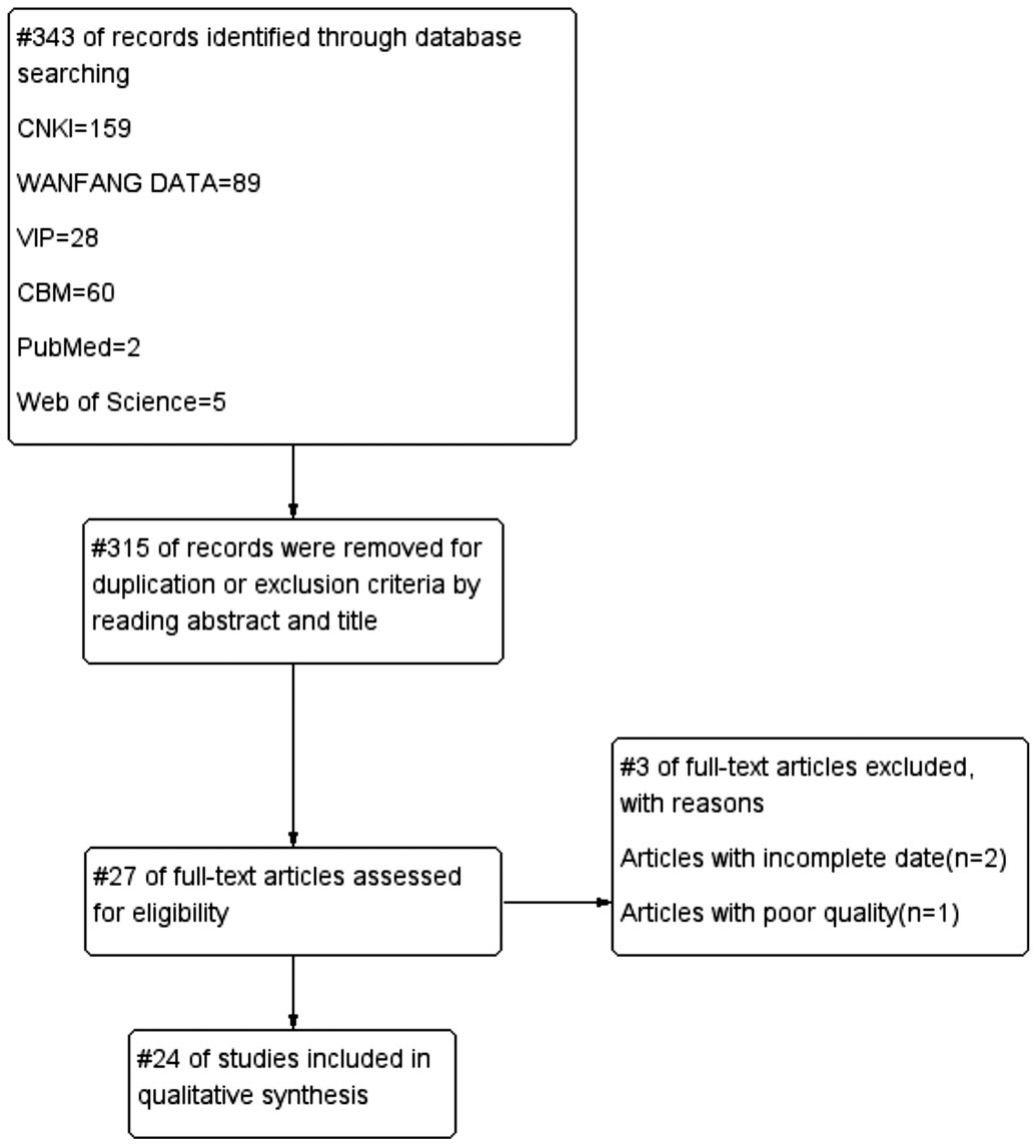
Flow diagram of the search method and selection process.

315 publications were retrieved according to the search strategy. Two researchers screened the articles by reading titles and abstracts to exclude duplicate literature. Twenty-eight articles were full text reviewed after screening, and 24 published articles were finally included. The detail of screening is shown in a flow diagram (Figure 1). Characteristics of included studies were summarized in Table 1. All 24 RCTs were published in Chinese, which involves 2039 subjects, with an average HAMD score of 29.99 before the treatment. The courses of treatment were all between two weeks and 12 weeks.

**Table 1.** Characteristics of included studies.

Literature included	Disease types	Study group cases (T)	Control group cases (C)	Average age (x ®±SD)		Sex (male/ female)		Interventions of study groups		Interventions of control group	Course (week)	Combined treatment	Outcome indicators ^Δ^
				T	C	T	C						
Cheng 2021	Poststroke depression	40	40	60.1 ± 5.3	62.1 ± 4.7	19/21	22/18	Modified CLMD +	Paroxetine /1dose QD	Paroxetine /1dose QD	8w	Basic treatment of stroke	①②
Ding and Gong 2021	Poststroke depression	48	48	59.1 ± 3.8	59.7 ± 3.5	27/21	26/22	Modified CLMD +	Paroxetine /10-40mg QD	Paroxetine /10-40mg QD	3w	Not mentioned	①②
Dong 2013	Poststroke depression	37	36	49.33 ± 7.81	50.26 ± 7.39	16/21	17/19	Modified CLMD +	Deanxit /1dose TID	Deanxit /1dose TID	4w	Basic treatment of stroke	①
Fu et al. 2021	Postpartum depression	69	69	28.6 ± 2.3	29. 5 ± 2. 4	0/69	0/69	Modified CLMD +	Paroxetine /20–50mg QD	Paroxetine /20–50mg QD	12w	Psychotherapy	①②④⑥
Gao and Zhang 2019	Poststroke depression	75	75	66.5 ± 11.3	69.5 ± 12.0	30/45	33/42	Modified CLMD +	Deanxit /1dose BID	Deanxit /1dose BID	3w	Basic treatment of stroke	①②③
Guo et al. 2019	Poststroke depression	41	41	56.3 ± 11.7	55.8 ± 12.4	26/15	28/13	Modified CLMD +	Fluoxetine /20mg QD	Fluoxetine /20mg QD		Not mentioned	①④
Han 2018	Poststroke depression	47	47	59.71 ± 5.29	59.69 ± 5.28	33/14	13119	Modified CLMD +	Fluoxetine /20mg QD	Fluoxetine /20mg QD	4w	Basic treatment of stroke	①④
Huang et al. 2016	Poststroke depression	38	40	58.53 ± 9.42	57.65 ± 9.38	21/17	23/17	Modified CLMD +	Deanxit /1dose QD	Deanxit /1dose QD	8w	Not mentioned	①②③④
Li 2017	Poststroke depression	35	35	63.52 ± 6.14	67.45 ± 6.14	19/16	44918	Modified CLMD +	Fluoxetine /20–40mg QD	Fluoxetine /20–40mg QD	8w	Basic treatment of stroke	①②
Li 2018	Poststroke depression	36	36	56.97 ± 10.83	57.06 ± 11.02	16/20	19/17	Modified CLMD +	Deanxit /1dose BID	Deanxit /1dose BID	4w	Basic treatment of stroke	①
Li 2010	Poststroke depression	36	34	56.8	59.6	17/19	15/19	Modified CLMD +	Fluoxetine /20mg QD	Fluoxetine /20mg QD	8w	Basic treatment of stroke	①②④
Lin et al. 2019	Perimenopause depression	30	30	50.63 ± 2.58	50.17 ± 2.72	0/30	0/30	Modified CLMD +	Paroxetine /20mg QD	Paroxetine /20mg QD	8w	Not mentioned	①②④
Liu 2016	Poststroke depression	40	40	55.21 ± 5.36	55.01 ± 5.22	44894	44923	Modified CLMD +	Deanxit /1dose BID	Deanxit /1dose BID	4w	Basic treatment of stroke	①
Liu and Yang 2017	Poststroke depression	48	47	48.35 ± 6.24	48.35 ± 6.24	26/22	26/21	Modified CLMD +	Paroxetine /10–40mg QD	Paroxetine /10–40mg QD	12w	Not mentioned	①③④
Liu 2021	Postpartum depression	34	34	25.10 ± 2.26	24.65 ± 2.30	0/34	0/34	Modified CLMD +	Paroxetine /20–50mg QD	Paroxetine /20–50mg QD	4w	Not mentioned	①④⑤⑥
Liu and Wang 2015	Poststroke depression	30	30	65.23 ± 7.41	68.45 ± 8.52	17/13	44854	Modified CLMD +	Deanxit /1dose BID→QD	Deanxit /1dose BID→QD	8w	Basic treatment of stroke	①②③
Luo and Wang 2019	Poststroke depression	35	35	58.69 ± 3.73	58.70 ± 3.69	44918	22/13	Modified CLMD +	Paroxetine /20–40mg QD	Paroxetine /20–40mg QD	4w	Basic treatment of stroke	①②⑦
Sun 2011	Poststroke depression	43	40	50.46 ± 6.37	54.35 ± 5.63	24/19	44923	Modified CLMD +	Paroxetine /20mg QD	Paroxetine /20mg QD	4w	Basic treatment of stroke	①②③
Wang et al. 2020	Depression with insomnia	30	28	50.2 ± 4.9	51.6 ± 4.7	44795	44823	Modified CLMD +	Escitalopram /10mg BID	Escitalopram /10mg BID	2w	Eszopiclone /1mg QN + Lorazepam /1mg QN	①②⑤
Wu 2017	Poststroke depression	41	41	59.3 ± 3.6		48/34		Modified CLMD +	Deanxit /1dose BID→QD	Deanxit /1dose BID→QD	8w	Basic treatment of stroke	①②③
Yan and Yang 2019	Poststroke depression	34	34	60.4 ± 3.42	60.22 ± 3.37	44917	44858	Modified CLMD +	Fluoxetine /20mg QD	Fluoxetine /20mg QD	8w	Basic treatment of stroke	①③④⑦
Yang 2018	Tumor-related depression	46	46	49.12 ± 7.96	48.86 ± 8.13	0/46	0/46	Modified CLMD +	Fluoxetine /20–40mg QD	Fluoxetine /20–40mg QD	4w	Not mentioned	①④
Zhang et al. 2022	Poststroke depression	45	45	63.82 ± 3.32	63.13 ± 3.21	25/20	24/21	Modified CLMD +	Sertraline /50mg QD	Sertraline /50mg QD	8w	Basic treatment of stroke	①③⑤⑥⑦
Zhang et al. 2017	Tumor-related depression	65	65	64.17 ± 5.67	65.03 ± 5.81	39/26	38/27	Modified CLMD +	Escitalopram /10mg QD	Escitalopram /10mg QD	8w	Basic treatment of cancer	①②④

ΔOutcome indicators: ①HAMD score. ②Total effective rate. ③NIHSS score. ④Adverse reaction rate. ⑤PSQI score. ⑥Monoamine transmitter. ⑦Cytokine level.

### Quality assessment in included studies

**Figure 2.**
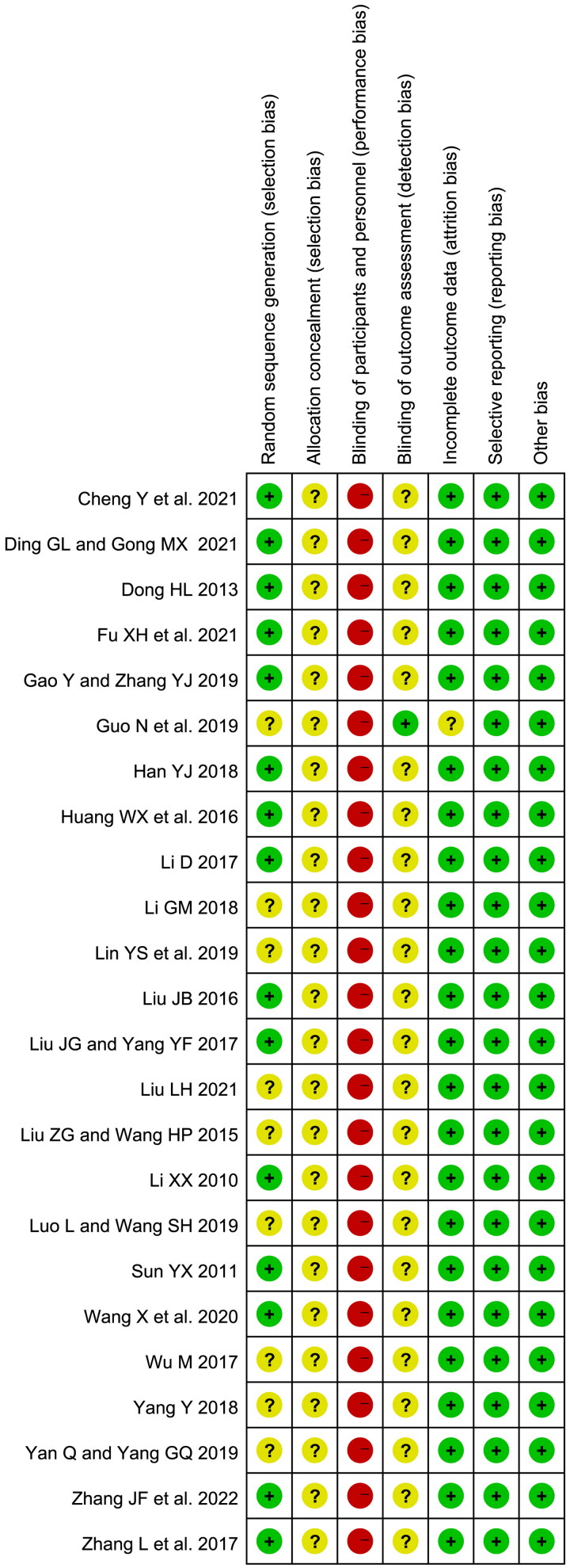
Risk of bias assessment.

Risk of bias was assessed (Figure 2) by two researchers. Fifteen included studies reported specific random sequence generation, and the rest were evaluated as unclear risk of bias. In terms of allocation concealment, all studies were marked as unclear risk. Due to the nature of TCM decoction, no studies described the blinding method, therefore all RCTs were rated as high risk in performance bias. Twenty-three studies were assessed as unclear risk in terms of blinding of outcome assessment. All studies were assessed as low risk of bias in selective reporting. There was no incomplete outcome data bias in all studies but one (Guo et al. 2019), which didn’t report the treatment course, evaluated as unclear risk.

### Meta-analysis results

#### Total effective rate

##### Analysis of total effective rate

**Figure 3.**
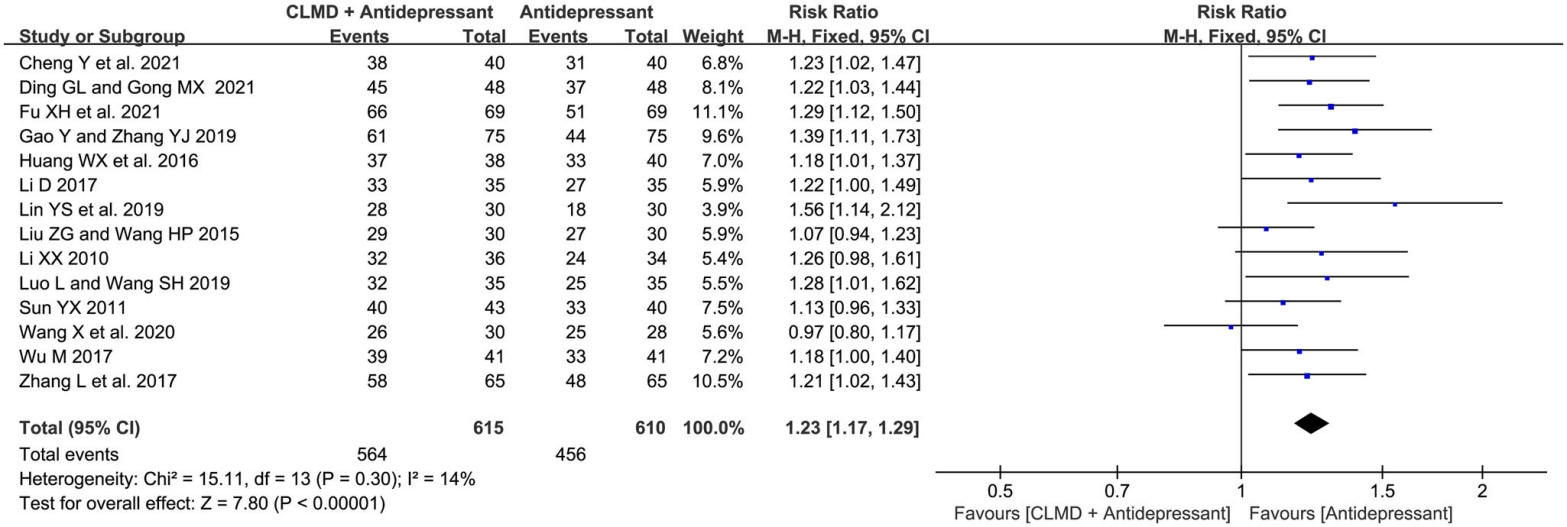
Forest plot of total effective rate.

Fourteen articles reported the total effective rate (Figure 3). Heterogeneity test showed that there was no significant heterogeneity between studies (*p* = 0.30, *I*^2^ = 14%), and a meta-analysis was conducted using fixed model. The result showed that the total effective rate of CLMD combined with antidepressants was higher than that of antidepressants alone, and the difference between two groups was statistically significant (RR = 1.23, 95%CI: 1.17 ∼ 1.29, *p* < 0.00001).

##### Comparison of studies with different courses

**Figure 4.**
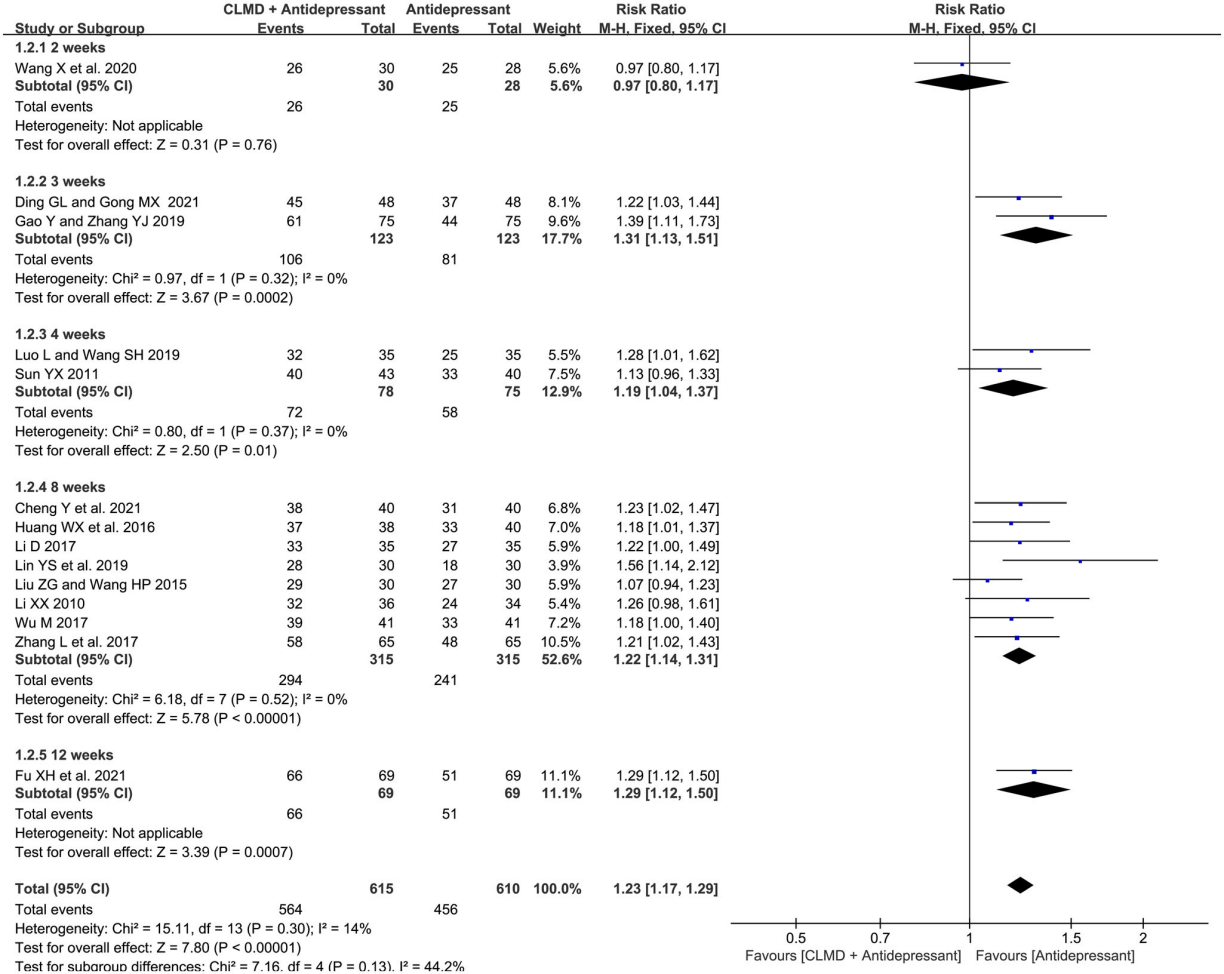
Forest plot of subgroups with different courses in terms of total effective rate.

We conducted subgroup analysis according to the course of treatment. All studies were divided into five subgroups (Figure 4): 2, 3, 4, 8, and 12 weeks. There was no significant heterogeneity between studies in 3-, 4-, and 8-week subgroups, and the fixed-effect model was used for meta-analysis. The result showed that the total effective rate of combined treatment was significantly higher than antidepressants alone with courses of 3-, 4-, and 8-week (RR = 1.31, 95%CI: 1.13 ∼ 1.51, *p* = 0.0002; RR = 1.19, 95%CI: 1.04 ∼ 1.37, *p* = 0.01; RR = 1.22, 95%CI: 1.14 ∼ 1.31, *p* < 0.00001), which indicated that CLMD combined group might have higher effective rate than control group when the treatment is 3 weeks or more.

##### Comparison of studies with different combination options

**Figure 5.**
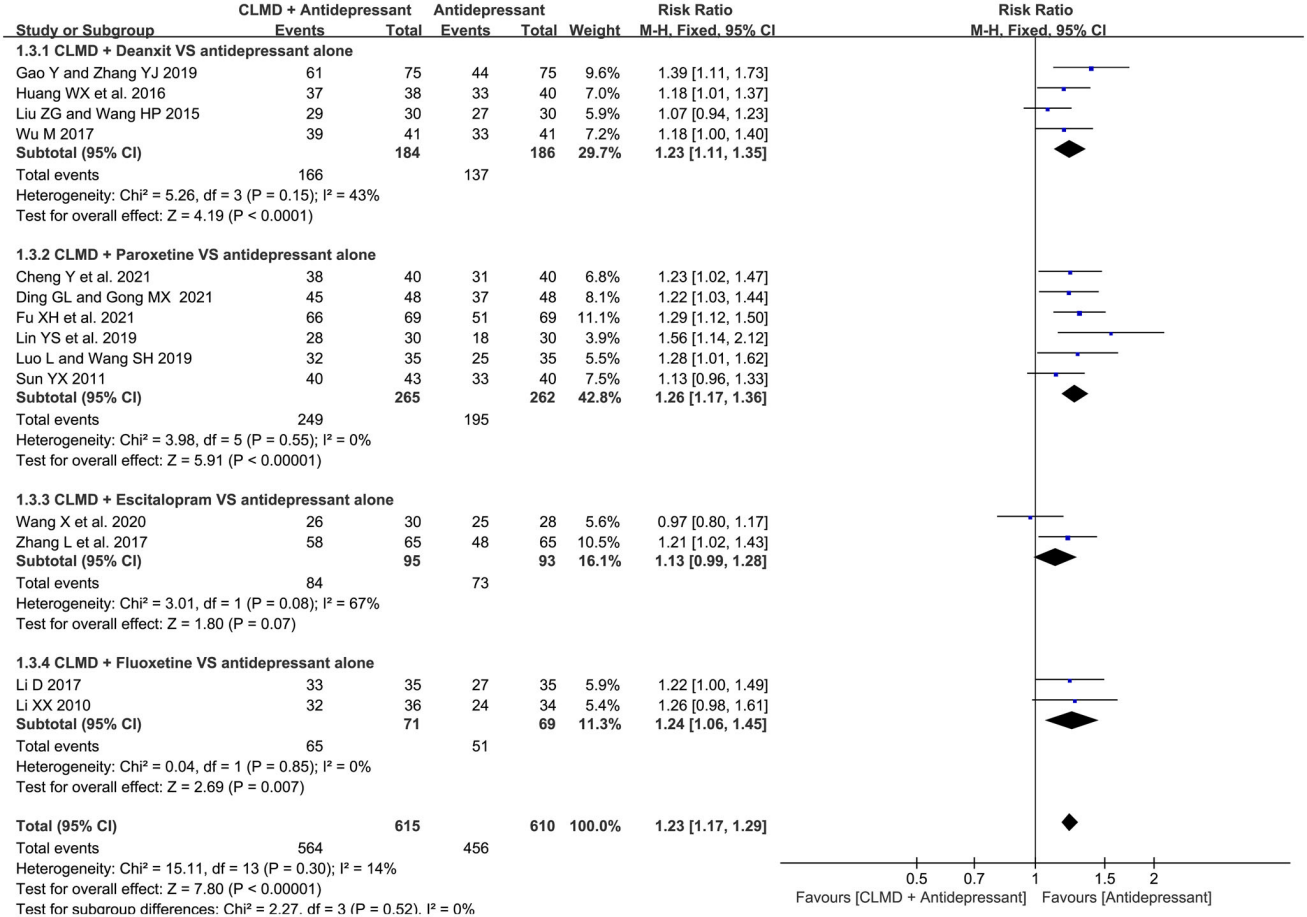
Forest plot of subgroups with different combined treatment in terms of total effective rate.

We conducted subgroup analysis according to different antidepressants combined (Figure 5). Studies were divided into four subgroups: deanxit, paroxetine, fluoxetine, escitalopram combined subgroups. There was high heterogeneity in escitalopram subgroup (*p* = 0.08, *I*^2^ = 67%). A fixed-effect model was used for meta-analysis. And the result showed that all combination options had significantly higher effective rate than control group except for escitalopram combined treatment (RR = 1.13, 95%CI: 0.99 ∼ 1.28, *p* = 0.07).

#### HAMD score

##### Analysis of HAMD score

**Figure 6.**
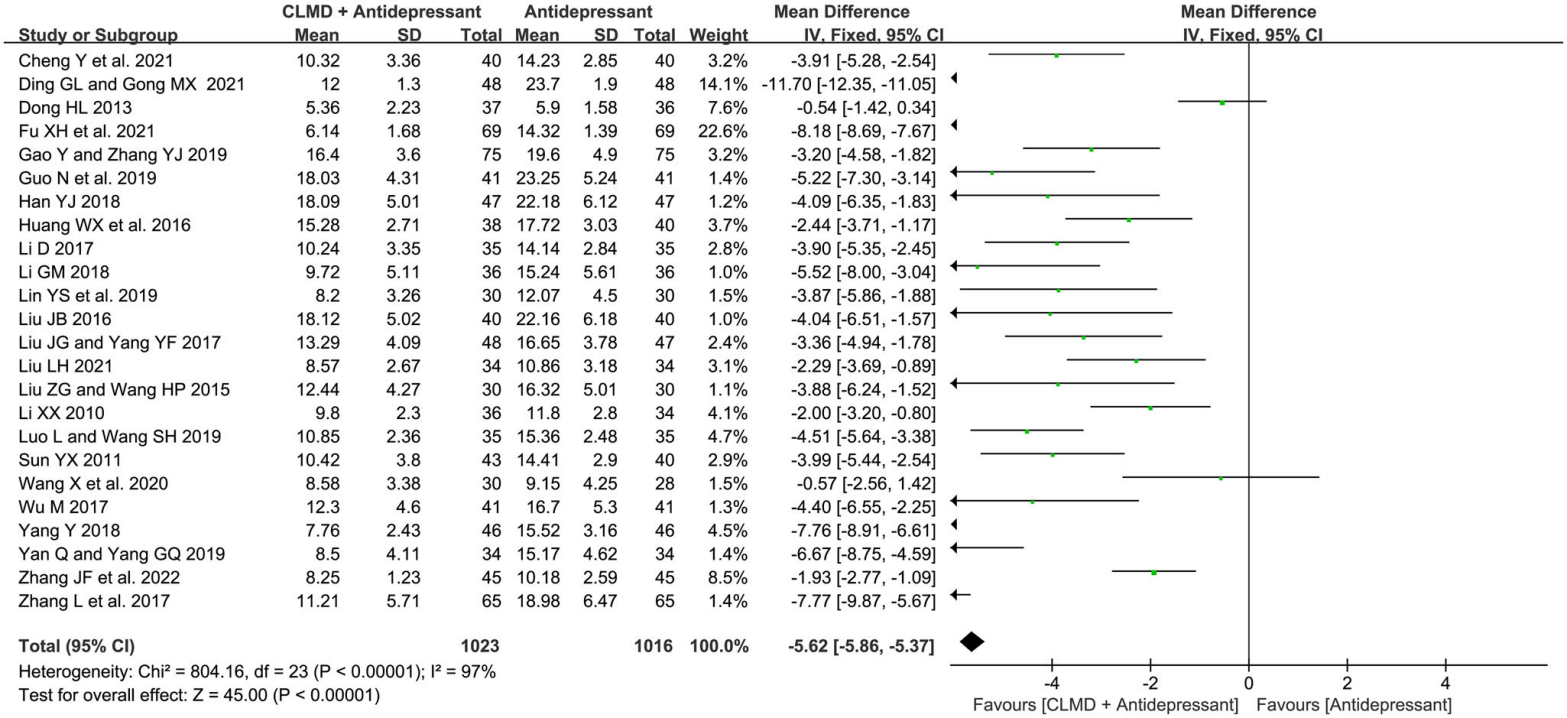
Forest plot of HAMD score.

**Figure 7.**
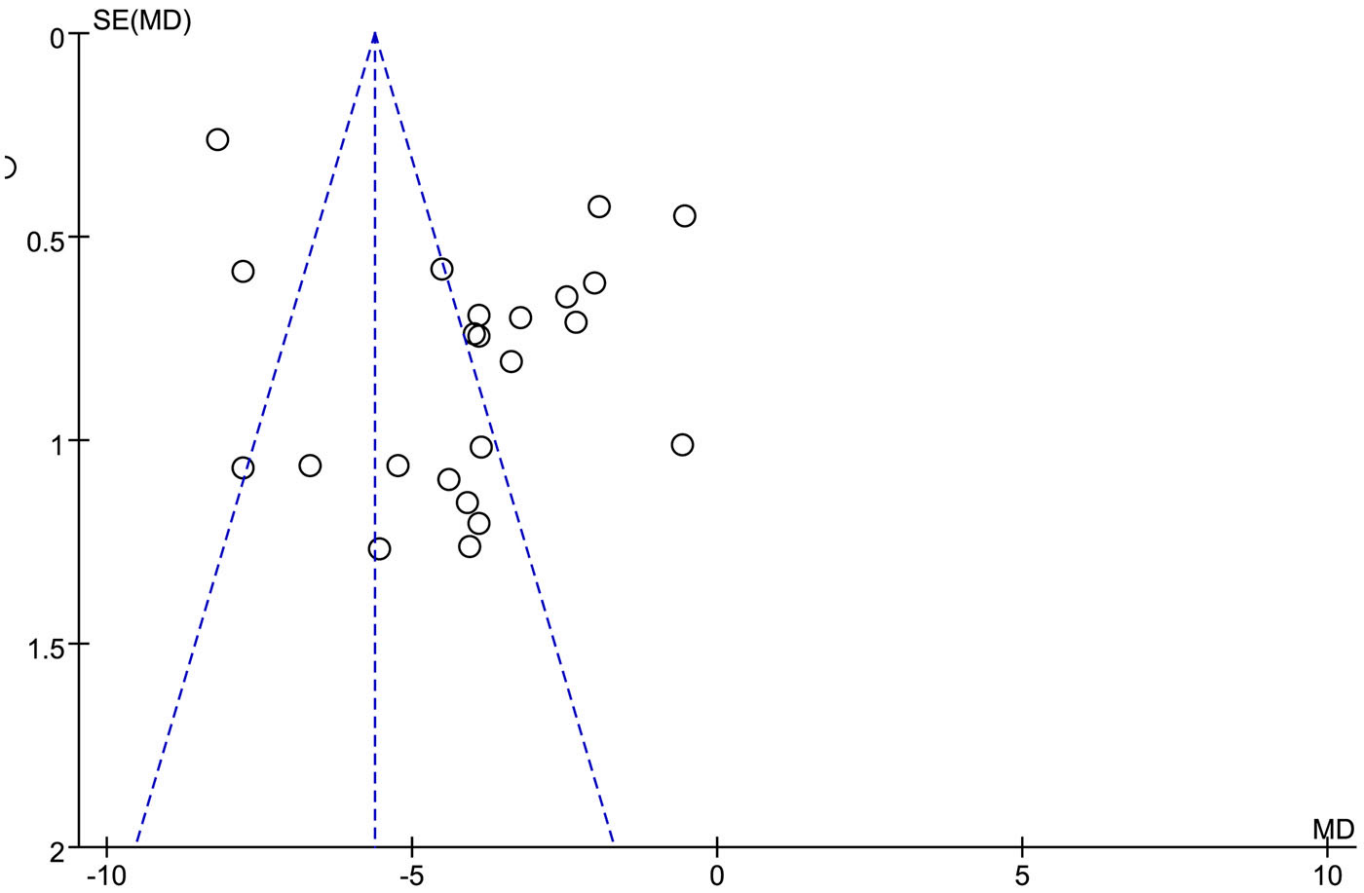
Funnel plot of HAMD score for detecting the publication bias.

All 24 articles reported the HAMD score, and the heterogeneity test showed that there was high heterogeneity among literature (*p* < 0.00001, *I*^2^ = 97%). The fixed-effect model was used for analysis. The result showed that the HAMD score of study group was lower than that of control group (Figure 6), and the difference was significant (MD = −5.62, 95%CI: −5.86 ∼ −5.37, *p* < 0.00001). A funnel plot was generated to detect the publication bias (Figure 7), which was not symmetrical in general, representing the certain publication bias.

##### Comparation of different comorbid disease

**Figure 8.**
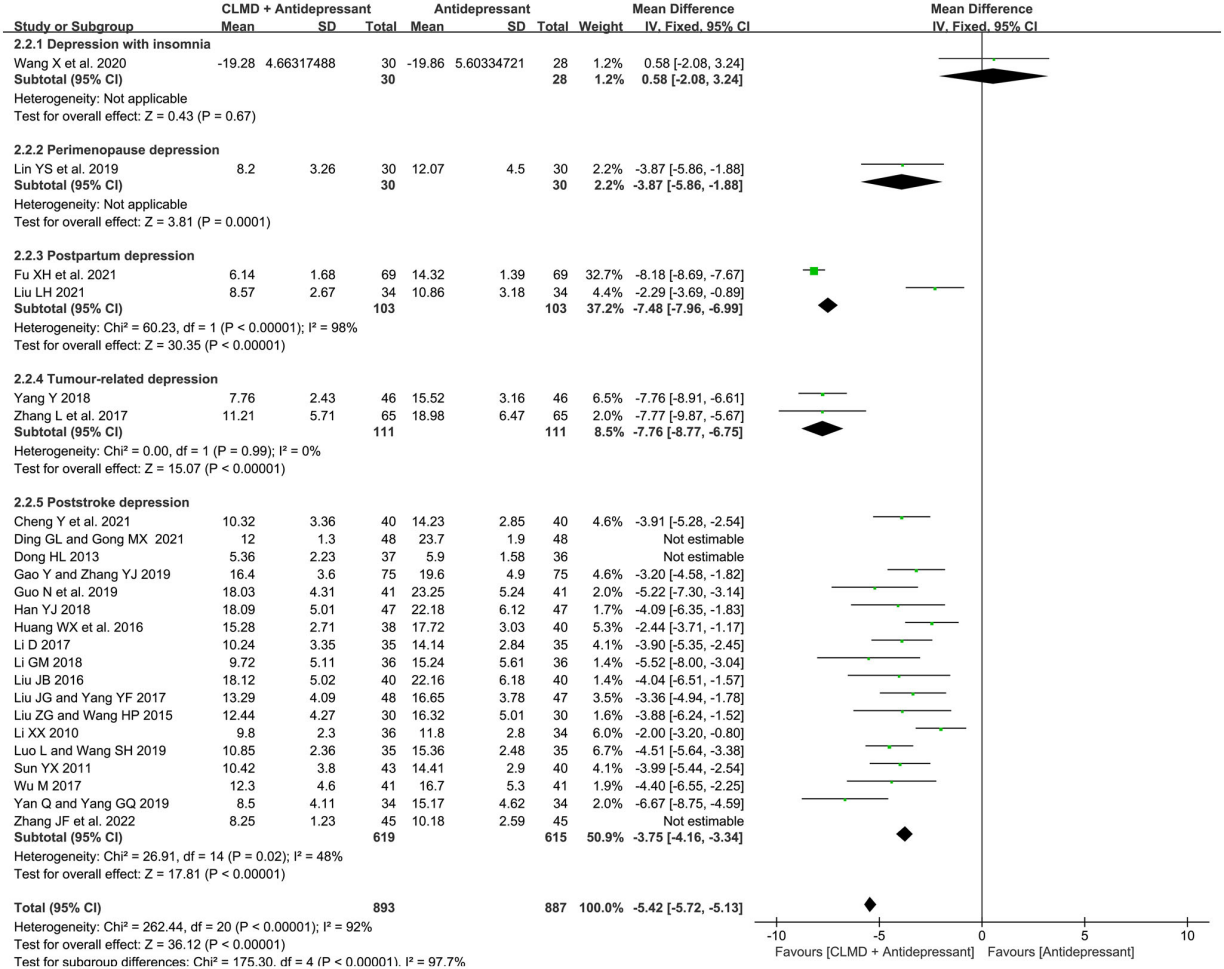
Forest plot of subgroups with different comorbid types in terms of HAMD score.

To explore the heterogeneity among studies, we conducted subgroup analysis according to different comorbid types. As is shown in Figure 8, included studies consisted of 5 types of comorbid diseases: depression with insomnia (1 article), postpartum depression (2 articles), perimenopause depression (1 article), tumor-related depression (2 articles) and poststroke depression (18 articles). There was high heterogeneity in postpartum depression subgroup (*p* < 0.00001, *I*^2^ = 98%), low heterogeneity in tumor-related depression subgroup (*p* = 0.99, *I*^2^ = 0%), and low heterogeneity in poststroke depression subgroup after three articles (Dong 2013; Ding and Gong 2021; Zhang et al. 2022) were omitted (*p* = 0.02, *I*^2^ = 48%). The fixed-effect model was used for analysis. Except for the subgroup of depression with insomnia and perimenopause depression which could not be analyzed for the quantity of literature, the HAMD score of study groups in other subgroups was significantly lower than that of the control groups, indicating that CLMD combined with antidepressants therapy has greater efficacy in treating MMD.

##### Timeline of HAMD score

**Figure 9.**
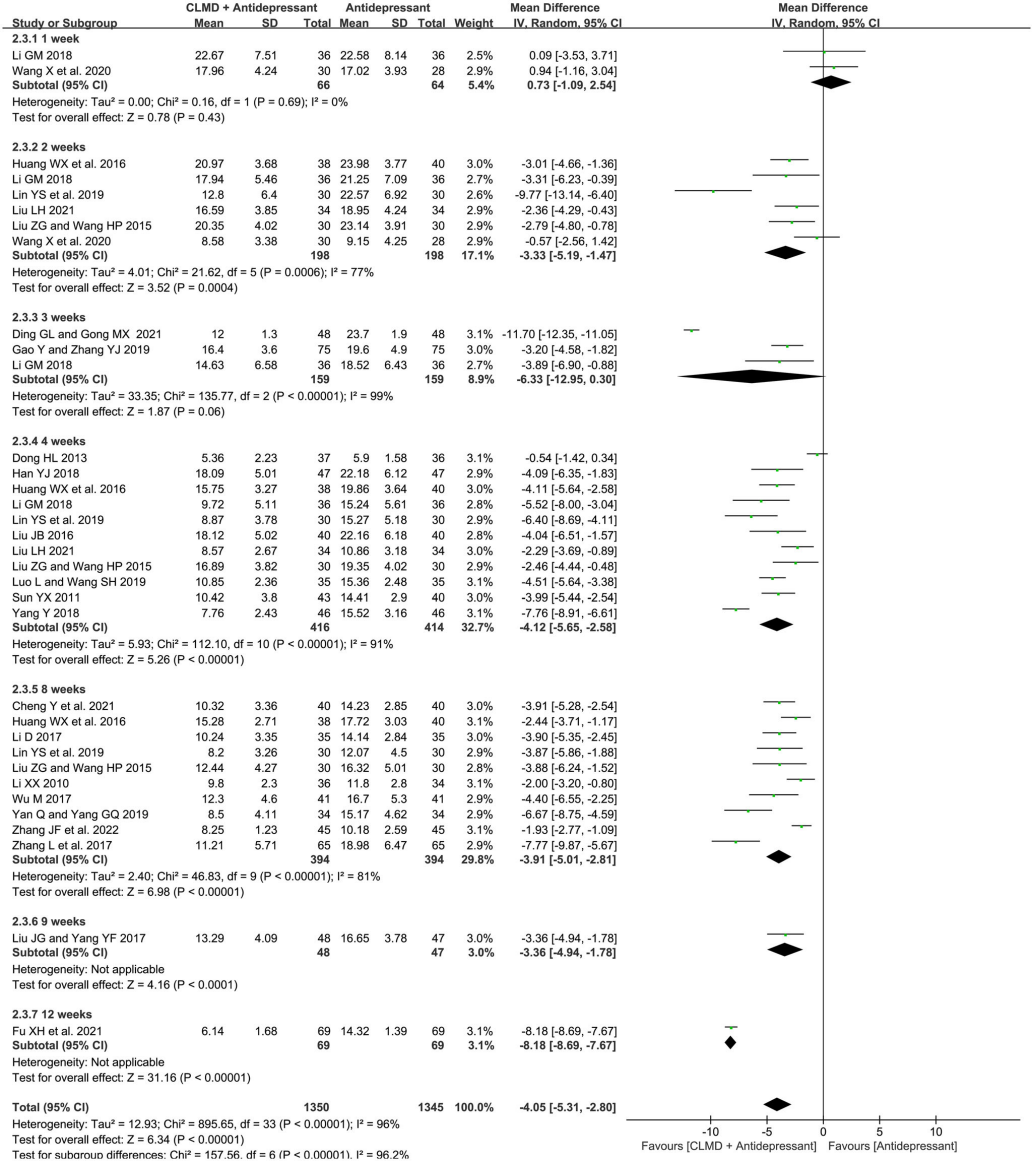
Forest plot of subgroups in timeline in terms of HAMD score.

We conducted the analysis of the timeline of HAMD score extracted from all included studies. As is shown in Figure 9, HAMD score was reported at 1st, 2nd, 3rd, 4th, 8th, 9th, and 12th week, respectively. Two studies reported the HAMD score at first week with no significant heterogeneity (*p* = 0.69, *I*^2^ = 0%). There was significant heterogeneity in 2nd, 3rd, 4th, 8th week subgroup (*p* = 0.006, *I*^2^ = 77%; *p* < 0.00001, *I*^2^ = 99%; *p* < 0.00001, *I*^2^ = 91%; *p* < 0.00001, *I*^2^ = 81%), and the random-effect model was used for analysis. There’s only one study reporting the HAMD score at both 9th and 12th week (Liu and Yang 2017; Fu et al. 2021), which could not be used for meta-analysis. Results showed that the HAMD score of study group at the 2nd, 4th and 8th week was significantly lower than that of control group (MD = −3.33, 95%CI: −5.19 ∼ −1.47, *p* = 0.0004; MD = −4.12, 95%CI: −5.65 ∼ −2.58, *p* < 0.00001; MD = −3.91, 95%CI: −5.01 ∼ −2.81, *p* < 0.00001), indicating that CLMD combined treatment had a positive clinical effect when the treatment course is longer than two weeks.

#### NIHSS score

**Figure 10.**
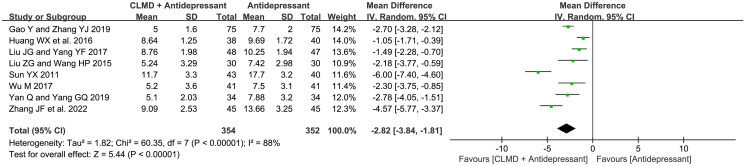
Forest plot of NIHSS score.

Eight RCTs reported a NIHSS score (Figure 10). The test presented high heterogeneity among studies (*p* < 0.00001, *I*^2^ = 88%), and the random-effect model was used for analysis. The result showed that the NIHSS score of CLMD combined group was significantly lower than control group (MD = −2.82, 95%CI: −3.84 ∼ −1.81, *p* < 0.00001).

#### PSQI score

**Figure 11.**
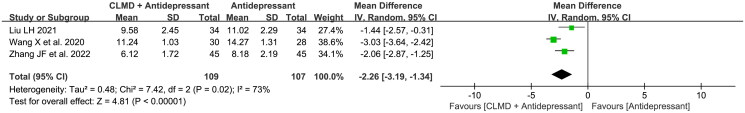
Forest plot of PSQI score.

Three RCTs reported a PSQI score (Figure 11). There was high heterogeneity among studies (*p* = 0.02, *I*^2^ = 73%). The random-effect model was used for analysis. As is shown in Figure 11, study group had a lower PSQI score than control group, and the difference was significant (MD = −2.26, 95%CI: −3.19 ∼ −1.34, *p* < 0.00001).

#### Monoamine transmitter level and cytokine level

Monoamine transmitter level and cytokine level were analyzed and summarized in Table 2.

**Table 2.** Monoamine transmitter and cytokine level.

Outcome indicators	Cases		Heterogeneity		Results		
	Study group	Control group	*I*^2^ (%)	*p*	Effect size index	95% CI	*p*
5-HT	148	148	95	<0.00001	SMD = 1.99	[0.68, 3.30]	0.003
NE	79	79	0	0.88	SMD = 1.99	[0.63, 1.29]	<0.00001
DA	79	79	0	0.94	SMD = 0.86	[0.54, 1.19]	<0.00001
TNF-α	69	69	100	<0.00001	MD = −27.37	[−57.80, 3.07]	0.08
IL-1β	79	79	97	<0.00001	MD = −14.64	[−24,41, −4.87]	0.003

Three studies reported the 5-HT level. The measurement units for this continuous variable among studies were different, therefore SMD was used for analysis. There was significant heterogeneity (*p* < 0.00001, *I*^2^ = 95%), the random-effect model was used for analysis. The result showed that CLMD combined group had significantly higher 5-HT level than single drug group (SMD = 1.99, 95%CI: 0.68 ∼ 3.30, *p* = 0.003).

Two studies reported the NE level. SMD and the fixed-effect model were used for analysis for low heterogeneity (*p* = 0.88, *I*^2^ = 0%) and different measurement units. The result showed that the NE level of study group was significantly higher than that of control group (SMD = 0.96, 95%CI: 0.63 ∼ 1.29, *p* < 0.00001).

Two studies reported the DA level. The data of two groups was analyzed using SMD. There was no significant heterogeneity (*p* = 0.94, *I*^2^ = 0%), the fixed-effect model was used for analysis. The result showed that the DA level of study group was significantly higher than that of control group (SMD = 0.86, 95%CI: 0.54 ∼ 1.19, *p* < 0.00001).

Two studies reported the TNF-α level. MD was used to estimate the effect size. The random-effect model was used for high heterogeneity (*p* < 0.00001, *I*^2^ = 100%). The result showed that there was no significant difference in TNF-α level between study group and control group (MD = −27.37, 95%CI: −57.80 ∼ 3.07, *p* = 0.08).

Two studies reported the IL-1β level. MD was used to estimate the effect size. The random-effect model was used for high heterogeneity (*p* < 0.00001, *I*^2^ = 97%). The result showed that the IL-1β level of study group was significantly lower than that of control group (MD = −14.64, 95%CI: −24.41 ∼ −4.87, *p* = 0.003).

#### Incidence of adverse reaction

**Figure 12.**
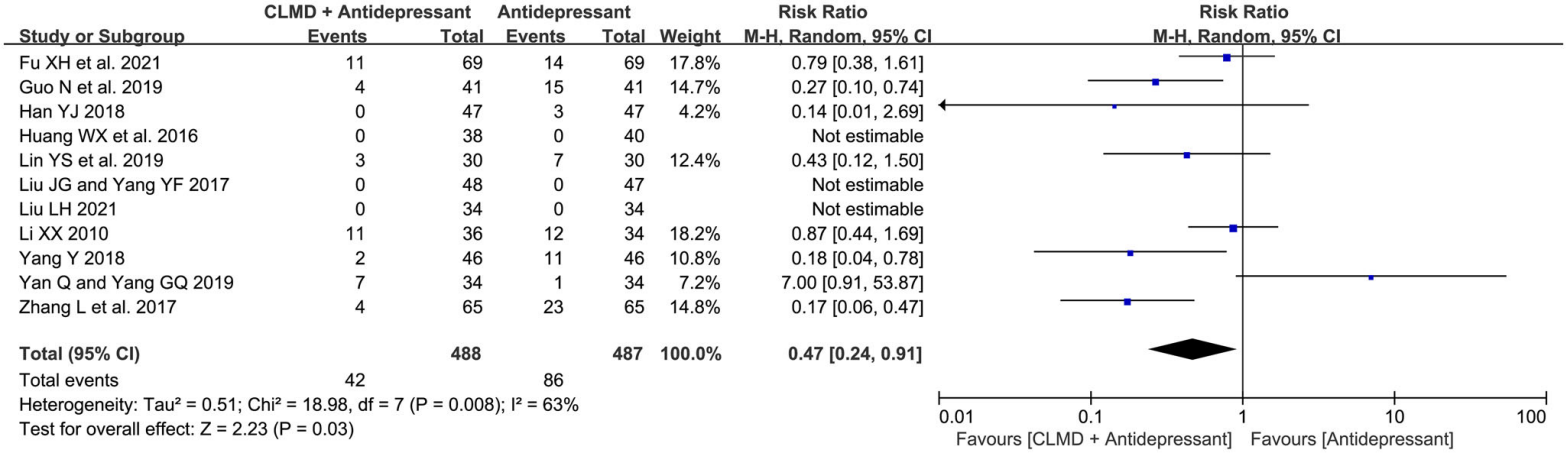
Forest plot of ADR incidence.

Eight RCTs reported the incidence of adverse reaction, of which three reported 0% ADR rate. The record information is summarized in Table 3. The main adverse reactions were gastrointestinal reactions and vegetative nervous disorders, of which gastrointestinal reactions were more common. A total of 488 cases in the study group and 487 cases in the control group were included in 8 articles. A total of 42 and 86 cases of adverse reactions were reported in study group and control group respectively. RR was used to estimate the effect size. The random-effect model was used for meta-analysis (Figure 12), and the result showed that the incidence of adverse reactions in the study group was significantly lower than that in the control group (RR = 0.47, 95%CI: 0.24 ∼ 0.91, *p* = 0.03).

**Table 3.** Record information of ADR collected from literature.

Literatures included	ADR records
Fu et al. 2021	Drowsiness, blurred vision, gastrointestinal reactions, dizziness, tremor, weight gain.
Guo et al. 2019	Paralysis, dysphagia, cognitive impairment, rectal dysfunction.
Han 2018	Insomnia.
Huang et al. 2016	(ADR incidence was reported as zero.)
Li 2018	Abnormal urine routine, blood routine, electrocardiogram, and liver function; Dizziness, palpitation, insomnia.
Lin et al. 2019	Nausea, anorexia, constipation, anorexia, dry mouth, dizziness.
Liu and Yang 2017	(ADR incidence was reported as zero.)
Liu 2021	(ADR incidence was reported as zero.)
Yan and Yang 2019	Anorexia, nausea, insomnia.
Yang 2018	Nausea and vomiting, headache, dizziness, insomnia, and fever.
Zhang et al. 2017	Dry mouth, drowsiness, headache, nausea.

### Sensitivity analysis

We conducted sensitivity analysis to assess the influence of each individual study on results by sequential omission of individual studies. The analysis results in the 3rd week were greatly influenced by single article. The difference of study and control group turned significant after Ding and Gong’s study (2021), or Gao and Zhang’s study (2019) were omitted. In analysis of the adverse reactions incidence, the results of a single study (Zhang et al. 2017; Han 2018; Yang 2018; Guo et al. 2019; Lin et al. 2019) could alter the significance of differences. The other results were less affected by sequential omission. Therefore, the sensitivity analysis confirmed that the results of this meta-analysis were generally statistically reliable and stable.

## Discussion

CLMD is a widely used compound TCM for depression, which is often combined with antidepressant medicine. Previous systematic review and meta-analysis of this formula for the treatment of depression combined with one single comorbid, such as stroke, cancer, menopausal syndrome, were numerous. But no systematic review and meta-analysis of comorbid depression of various diseases were reported. In this study, we evaluated the clinical effect of CLMD in treatment of various common comorbid depression by systematic review and meta-analysis to explore the clinical application of CLMD in MMD.

Among 24 included publications, studies on stroke and cancer complicating depression account for the majority. Stroke can lead to depression by causing damage to neural majority, while several types of cancer are closely related to long-term stress and depression, e.g., breast cancer, gastric cancer, bowel cancer and other malignant tumors, in which immune destruction is an important mechanism (Eckerling et al. 2021; Rudak et al. 2021). In addition, cancer patients have a high incidence of psychological disorders. The study of Chu et al. (2021) revealed that the incidence of depression and anxiety among cancer patients in China is 54.9 and 49.7%, respectively, and the suicide rate of cancer patients in China is nearly double that in Europe and America (63.17 per 100,000 people). Therefore, the evaluation and consideration of psychological status should be included in the treatment plan in the treatment of stroke and cancer patients. Both the disease and mental condition of patients should be taken into consideration to achieve the unity of mind and body.

It can also be noticed that SSRIs were used in 17 of 24 RCTs, which was consistent with the findings that the SSRI drugs, including escitalopram, fluoxetine, and paroxetine, shows significant efficacy and acceptability in double-blind RCTs (Cipriani et al. 2018).

Meta-analysis of the effective rate in different comorbid types treatment revealed that CLMD combined with antidepressants has a significantly higher effective rate than antidepressants alone, which shows the curative effect advantage of Chinese and western combined treatment. The addition of TCM in the treatment didn’t cause resistance or reduce treatment compliance, which indicated that TCM can be a routine choice in treating MMD, rather than an optional one. We didn’t find the advantage of antidepressant effect on early onset in combination. Rapid action is the primary task in the treatment of depression, and the average onset of SSRIs is more than two weeks. Generally, the rapid onset of an antidepressant is defined as an overall improvement in depressive symptoms within two weeks, or one week. Rapid antidepressant can not only improve the patient’s state as soon as possible, but also obtain a good treatment experience. In addition, it can avoid the risk of suicide in depression and have a good long-term prognosis, so rapid onset of antidepressant is significant. Therefore, the current clinical antidepressant treatment often combines antidepressants with different mechanisms, or reaches the maximum dose in a short period of time to improve depression. The studies included all reported the combination of CLMD and one single antidepressant. Because of the existing comorbidity, the drug dosage did not reach the saturated dose in a short time, but was regular dose, which was more conservative than the treatment of major depression. Therefore, most of treatment courses in studies were long. And the advantage can only be captured after three weeks, which didn’t reflect the rapid antidepressant effect. Our analysis suggests that, firstly, it may be due to the lack of efficacy evaluation at the 1st and 2nd week during treatment in included studies, which may be considered as defect in study design. Secondly, the criterions of efficacy evaluation were different, which also leaded to bias. In addition, in the meta-analysis of HAMD score, we found that the CLMD combined treatment performed antidepressant effects after two weeks, which suggests that the combination of CLMD does not shorten the onset of antidepressants.

In this study, we also made a preliminary analysis of the combination of antidepressants with different mechanisms. We found that CLMD significantly increased the total effective rate when combined with deanxit, paroxetine, fluoxetine, except for escitalopram. As a commonly used new antidepressant in recent years, escitalopram may mask the antidepressant effect of CLMD for its clinical efficacy advantage, for which the advantage of combination with CLMD was not shown. HAMD score is one of the major outcome indicators of efficacy evaluation. Meta-analysis showed that HAMD score of CLMD combination group was significantly lower than that of control group, indicating that the combination of CLMD and most antidepressants can enhance the clinical effect.

In terms of multimorbidity treatment, several included studies reported NIHSS and PSQI for neurological function and sleeping quality evaluation, respectively. We analyzed the influence of combination therapy on neurological function in stroke and sleeping quality in insomnia. HAMD score was highly correlated with NIHSS score in patients with poststroke depression (PSD) (Li and Ren 2014). With the increase of NIHSS score of patients, the disease of PSD tended to worsen. The meta-analysis showed that compared with control group, CLMD can significantly reduce NIHSS score of stroke patients, and has positive significance for promoting neurological rehabilitation. Insomnia is a common symptom of depression, and also be studied as an independent disease. Recent studies (Taylor et al. 2005) have found that insomnia is closely related to the occurrence of depression. Insomnia and depression may share the same mechanism. People with insomnia are 9.82 times more likely to suffer from depression than normal people. Improving patients’ sleeping quality plays an important role in the treatment of depression. PSQI score (Lu et al. 2014) is a reference for clinical evaluation of the efficacy of insomnia treatment and comparison of intervention measures. Meta-analysis showed that CLMD combined with antidepressants markedly decreased PSQI score of patients with insomnia, which can improve the life quality of patients with depression.

Although the biological mechanism of depression is still unknown and there are no specific biomarkers for biological diagnosis, serum monoamine transmitter and cytokine level are commonly used in clinical objective evaluation indicators of depression. The level of the inflammatory marker IL-6 and TNF-α in serum are significantly higher in depressed patients than in non-depressed individuals (Savitz et al. 2018). The inflammatory marker is important parameters to guide the treatment of depression (Ferenczi et al. 2016). It was reported (Li 2015) that CLMD combined with venlafaxine can significantly reduce IL-6, IL-1β and TNF-α level in elderly women with depression. This study shows that the combination of CLMD and antidepressants improved 5-HT and DA level while reducing the cytokine level.

Clinical application of SSRIs, SNRIs and TCAs has a high risk of adverse reactions, and is easy to cause drug resistance and drug dependence. In terms of adverse reactions, we summarized ADR records from literature and analyzed the incidence of ADR. The results showed that the incidence of ADR in study group was significantly lower than that in control group. Adverse reactions in studies mainly involved gastrointestinal reactions and autonomic dysfunction, among which gastrointestinal reactions were the most common adverse reactions. As a compound TCM, one of the effects of CLMD is to adjust gastrointestinal peristalsis and improve gastrointestinal function, which has natural advantages in reducing gastrointestinal ADRs. Previous study (Wan et al. 2021) showed that CLMD combined with antidepressants has significantly higher safety than antidepressants alone.

## Conclusions

This study has found that the combination of CLMD with SSRI antidepressants showed significantly higher clinical efficacy and safety than antidepressants alone. The combination of CLMD presented enhancing antidepressant potential two weeks after the treatment. When it comes to total effective rate, the combination has advantages in both anti-depression and treating the protopathy diseases. However, due to the limited amount of literature included in this study, there was certain bias in the selection of multimorbidity types and drug combinations, and the efficacy evaluation were somewhat subjective, which may lead to deviation of research conclusions. In the future, more standardized and high-quality clinical studies are needed to explore the antidepression potential of TCM, and to provide strong evidence for the treatment of MMD by integrated traditional Chinese and western medicine.

## Data Availability

The data used to support the results of this study are available from the corresponding authors upon request.

## References

[CIT0001] Chinese Society of Psychiatry. 2001. The Chinese classification and diagnostic criteria for mental disorders, 3rd edition. Chin J Psychiatry. 34(03):59–63.

[CIT0002] Cheng Y. 2021. Influence of Chaihu Longgu Muli decoction on CSS score in patients with post-stroke depression. China Pract Med. 16(26):156–158.

[CIT0003] Chu Q, Cheong IH, Le PD, Yang LH, Wang H, Hall BJ. 2021. The unaddressed mental health burden among cancer patients in China: a call to action. Lancet Psychiatry. 8(8):646–647. doi: 10.1016/S2215-0366(21)00180-2. 34303401

[CIT0004] Cipriani A, Furukawa TA, Salanti G, Chaimani A, Atkinson LZ, Ogawa Y, Leucht S, Ruhe HG, Turner EH, Higgins JPT, et al. 2018. Comparative efficacy and acceptability of 21 antidepressant drugs for the acute treatment of adults with major depressive disorder: a systematic review and network meta-analysis. Lancet. 391(10128):1357–1366. doi: 10.1016/S0140-6736(17)32802-7. 29477251 PMC5889788

[CIT0005] Dahl J, Ormstad H, Aass HC, Malt UF, Bendz LT, Sandvik L, Brundin L, Andreassen OA. 2014. The plasma levels of various cytokines are increased during ongoing depression and are reduced to normal levels after recovery. Psychoneuroendocrinology. 45:77–86. doi: 10.1016/j.psyneuen.2014.03.019. 24845179

[CIT0006] Dean J, Keshavan M. 2017. The neurobiology of depression: an integrated view. Asian J Psychiatr. 27:101–111. doi: 10.1016/j.ajp.2017.01.025. 28558878

[CIT0007] Ding GL, Gong MX. 2021. Clinical efficacy of Chaihu and Longgu Muli decoction in treatment of patients with post-stroke depression and its influence on quality of life. Med Innov China. 18(26):82–86.

[CIT0008] Dong HL. 2013. Clinical research of Chaihu Jia Longgu Muli decoction for the treatment of post stroke depression. Acta Chin Med. 28:251–252.

[CIT0009] Eckerling A, Ricon-Becker I, Sorski L, Sandbank E, Ben-Eliyahu S. 2021. Stress and cancer: mechanisms, significance and future directions. Nat Rev Cancer. 21(12):767–785. doi: 10.1038/s41568-021-00395-5. 34508247

[CIT0010] Ferenczi EA, Zalocusky KA, Liston C, Grosenick L, Warden MR, Amatya D, Katovich K, Mehta H, Patenaude B, Ramakrishnan C, et al. 2016. Prefrontal cortical regulation of brainwide circuit dynamics and reward-related behavior. Science. 351(6268):aac9698. doi: 10.1126/science.aac9698. 26722001 PMC4772156

[CIT0011] Fu XH, Fu HG, Liang L, Kang RG. 2021. Curative effect of Chaihu Jia Longgu Muli decoction combined with paroxetine in the treatment of postpartum depression and its effect on serotonin. Mod J Integr Tradit Chin West Med. 30:188–191.

[CIT0012] Gao Y, Zhang YJ. 2019. The clinical trials of Chaihu-Longgu-Muli decoction combined flupentixol and melitracen tablets for the post-stroke depression. Int J Tradit Chin Med. 41:447–450.

[CIT0013] Guo N, Chen MR, Cui XC. 2019. Efficacy of Chaihu Jia Longgu Muli decoction combined with fluoxetine in the treatment of post-stroke depression. Chin J Mod Drug Appl. 13(11):159–160.

[CIT0014] Guo WF, Cao XL, Sheng L, Li JX, Zhang LK, Ma YZ. 2020. Expert consensus on the diagnosis and treatment of depression with integrated Chinese and western medicine. Chin J Integr Tradit West Med. 40:141–148.

[CIT0015] Han YJ. 2018. Chaihu plus Longgu Muli decoction combined with fluoxetine in the treatment of post stroke depression (Ganyu Tanrao) randomized parallel control study. J Pract Tradit Chin Intern Med. 32(09):47–50.

[CIT0016] Higgins JPT, Thomas J, Chandler J, Cumpston M, Li T, Page MJ, Welch VA, editors. 2022. Cochrane handbook for systematic reviews of interventions version 6.3 [updated February 2022]. Cochrane, 2022. www.training.cochrane.org/handbook.

[CIT0017] Huang WX, Tang JM, Wang WB. 2016. Clinical effect analysis of Chaihu Jia Longgu Muli decoction combined with Deanxit in the treatment of post-stroke depression. Contemp Med. 22(14):152–153.

[CIT0018] Li D. 2017. Clinical observation of Chaihu plus Longgu Muli decoction treatment of post-stroke depression. Med J Chin Peoples Health. 29(17):74–76.

[CIT0019] Li GQ, Ren CH. 2014. A study on the relationship between the prevalence of post-stroke depression and NIHSS, BI and MMSE scores. Mod J Integr Tradit Chin West Med. 23:3339–3341.

[CIT0020] Li GM. 2018. Study on Chaihu and Longgu Muli decoction combined with Deanxit in the treatment of post-stroke anxiety and depression. Pract Clin J Integr Tradit Chin West Med. 18(11):59–61.

[CIT0021] Li J. 2015. Study on the anti-depression effect of Chaihu Jia Longgu Muli decoction in the past five years. Clin J Tradit Chin Med. 27:1175–1178.

[CIT0022] Li XX. 2010. Clinical observation of post stroke depression on treatment with Chaihu plus Longggu Muli tang associated fluxetine hydrochloride. Mod Hosp. 10(04):73–74.

[CIT0023] Lin YS, Wang LY, Zhang ZY. 2019. Clinical effects observation of supplemented Chaihu Jia Longgu Muli decoction combined with paroxetine on perimenopause depression. China J Tradit Chin Med Pharm. 34:3330–3333.

[CIT0024] Liu HL, Wei FX, Qin XM, Liu XJ. 2020. Research progress in combination applications of antidepressant drugs. Chin J Chin Mater Med. 45:3776–3783. 10.19540/j.cnki.cjcmm.20200509.601 32893570

[CIT0025] Liu JB. 2016. Research the effect of Chaihu Longgu Muli decoction with antidepressant therapy in depression after stroke with kidney Yin deficiency syndrome. Shaanxi J Tradit Chin Med. 37:155–158.

[CIT0026] Liu JG, Yang YF. 2017. A clinical analysis of treating 48 cases of post-stroke depression with the Chaihu Longgu Muli decoction. Clin J Chin Med. 9(07):62–64.

[CIT0027] Liu LH. 2021. Clinical effect of using Chaihu Jia Longgu Muli decoction combined with western medicine in the treatment of postpartum depression patients and its influence on NE,5-HT. J Sichuan Tradit Chin Med. 39(01):162–165.

[CIT0028] Liu ZG, Wang HP. 2015. 30 Cases of post-stroke depression treated with Chaihu and Longgu Muli decoction and Deanxit. Jiangxi J Tradit Chin Med. 46(05):39–40.

[CIT0029] Lu TY, Li Y, Xia P, Zhang GQ, Wu DR. 2014. Analysis on reliability and validity of the Pittsburgh sleep quality index. Chongqing Med. 43:260–263.

[CIT0030] Luo L, Wang SH. 2019. Observation on the effect of Chaihu Jia Longgu Muli decoction on post-stroke depression. Chin J Clin Ration Drug Use. 12(28):42–43.

[CIT0031] Moussavi S, Chatterji S, Verdes E, Tandon A, Patel V, Ustun B. 2007. Depression, chronic diseases, and decrements in health: results from the world health surveys. Lancet. 370(9590):851–858. doi: 10.1016/S0140-6736(07)61415-9. 17826170

[CIT0032] Page MJ, McKenzie JE, Bossuyt PM, Boutron I, Hoffmann TC, Mulrow CD, Shamseer L, Tetzlaff JM, Akl EA, Brennan SE, et al. 2021. The PRISMA 2020 statement: an updated guideline for reporting systematic reviews. BMJ. 372:n71. doi: 10.1136/bmj.n71. 33782057 PMC8005924

[CIT0033] Pietzner M, Stewart ID, Raffler J, Khaw KT, Michelotti GA, Kastenmüller G, Wareham NJ, Langenberg C. 2021. Plasma metabolites to profile pathways in noncommunicable disease multimorbidity. Nat Med. 27(3):471–479. doi: 10.1038/s41591-021-01266-0. 33707775 PMC8127079

[CIT0034] Read JR, Sharpe L, Modini M, Dear BF. 2017. Multimorbidity and depression: a systematic review and meta-analysis. J Affect Disord. 221:36–46. doi: 10.1016/j.jad.2017.06.009. 28628766

[CIT0035] Rudak PT, Choi J, Parkins KM, Summers KL, Jackson DN, Foster PJ, Skaro AI, Leslie K, McAlister VC, Kuchroo VK, et al. 2021. Chronic stress physically spares but functionally impairs innate-like invariant T cells. Cell Rep. 35(2):108979. doi: 10.1016/j.celrep.2021.108979. 33852855 PMC8112805

[CIT0036] Savitz JB, Teague TK, Misaki M, Macaluso M, Wurfel BE, Meyer M, Drevets D, Yates W, Gleason O, Drevets WC, et al. 2018. Treatment of bipolar depression with minocycline and/or aspirin: an adaptive, 2 × 2 double-blind, randomized, placebo-controlled, phase IIA clinical trial. Transl Psychiatry. 8(1):27. doi: 10.1038/s41398-017-0073-7. 29362444 PMC5802452

[CIT0037] Sun YX. 2011. Clinical research of combination of Radix Bupleuri and Keel Oyster decoction with paroxetine in treating 43 patients with post-stroke depression. J Zhejiang Chin Med Univ. 35:332–333.

[CIT0038] Taylor DJ, Lichstein KL, Durrence HH, Reidel BW, Bush AJ. 2005. Epidemiology of insomnia, depression, and anxiety. Sleep. 28(11):1457–1464. doi: 10.1093/sleep/28.11.1457. 16335332

[CIT0039] Wan R, Song R, Fan Y, Li L, Zhang J, Zhang B, Li X, Wang S. 2021. Efficacy and safety of Chaihu Jia Longgu Muli decoction in the treatment of poststroke depression: a systematic review and meta-analysis. Evid Based Complement Alternat Med. 2021:7604537. doi: 10.1155/2021/7604537. 34457030 PMC8397549

[CIT0040] Wang X, Chen J, Zhang H, Huang Z, Zou Z, Chen Y, Sheng L, Xue W, Tang J, Wu H, et al. 2019. Immediate and persistent antidepressant-like effects of Chaihu-jia-Longgu-Muli-tang are associated with instantly up-regulated BDNF in the hippocampus of mice. Biosci Rep. 39(1):BSR20181539. 30473537 10.1042/BSR20181539 PMC6328878

[CIT0041] Wang X, Zou Z, Shen Q, Huang Z, Chen J, Tang J, Xue W, Tao W, Wu H, Wang D, et al. 2019. Involvement of NMDA-AKT-mTOR signaling in rapid antidepressant-like activity of Chaihu-jia-Longgu-Muli-tang on olfactory bulbectomized mice. Front Pharmacol. 9:1537. doi: 10.3389/fphar.2018.01537. 30687098 PMC6333740

[CIT0042] Wang X, Chen J, Yang N, Qiao HF. 2020. Clinical study of Chaihu Jia Longgu Muli decoction combined with western medicine in the treatment of 30 cases of severe depression with insomnia. Jiangsu J Tradit Chin Med. 52(10):27–29.

[CIT0043] Wu M. 2017. Observation on the therapeutic effect of Chaihu and Longgu Muli decoction combined with Deanxit in the treatment of post-stroke depression. Guaming J Chin Med. 32:1600–1602.

[CIT0044] Yan Q, Yang GQ. 2019. Effect of western medicine combined with Chaihu plus Longgu Muli decoction in the treatment of patients with post-stroke depression. Clin Res Pract. 4(36):152–154.

[CIT0045] Yang Y. 2018. The application of Chaihu Longgu Muli decoction in the treatment of breast cancer complicated with depression. Chin Med Mod Distance Educ China. 16(21):113–114.

[CIT0046] Zhang JF, Fan Y, Guo CJ, Song HQ. 2022. Effect of Chaihu Plus Longgu Muli decoction on monoamine neurotransmitters, amino acid neurotransmitters and inflammatory factors in patients with post-stoke depression. Inf Tradit Chin Med. 39(07):54–58.

[CIT0047] Zhang L, Wang JH, Wang JH, Yang JQ, Li XL. 2017. Clinical observation of post-tumor depression treated with Chaihu Jia Longgu Muli Tang and escitalopram. World J Integr Tradit West Med. 12:223–225.

